# Knockdown resistance mutations predict DDT resistance and pyrethroid tolerance in the visceral leishmaniasis vector *Phlebotomus argentipes*

**DOI:** 10.1371/journal.pntd.0005504

**Published:** 2017-04-17

**Authors:** Bruno Gomes, Bidyut Purkait, Rinki Michelle Deb, Aarti Rama, Rudra Pratap Singh, Geraldine Marie Foster, Michael Coleman, Vijay Kumar, Mark Paine, Pradeep Das, David Weetman

**Affiliations:** 1 Department of Vector Biology, Liverpool School of Tropical Medicine, Liverpool, United Kingdom; 2 Rajendra Memorial Research Institute of Medical Sciences (Indian Council of Medical Research), Agamkuan, Patna, Bihar, India; Mahidol University, THAILAND

## Abstract

**Background:**

Indoor residual spraying (IRS) with DDT has been the primary strategy for control of the visceral leishmaniasis (VL) vector *Phlebotomus argentipes* in India but efficacy may be compromised by resistance. Synthetic pyrethroids are now being introduced for IRS, but with a shared target site, the *para* voltage-gated sodium channel (VGSC), mutations affecting both insecticide classes could provide cross-resistance and represent a threat to sustainable IRS-based disease control.

**Methodology/Principal findings:**

A region of the *Vgsc* gene was sequenced in *P*. *argentipes* from the VL hotspot of Bihar, India. Two knockdown resistance (*kdr*) mutations were detected at codon 1014 (L1014F and L1014S), each common in mosquitoes, but previously unknown in phlebotomines. Both *kdr* mutations appear largely recessive, but as homozygotes (especially 1014F/F) or as 1014F/S heterozygotes exert a strong effect on DDT resistance, and significantly predict survivorship to class II pyrethroids in short-duration bioassays. The mutations are present at high frequency in wild *P*. *argentipes* populations from Bihar, with 1014F significantly more common in higher VL areas.

**Conclusions/Significance:**

The *Vgsc* mutations detected appear to be a primary mechanism underlying DDT resistance in *P*. *argentipes* and a contributory factor in reduced pyrethroid susceptibility, suggesting a potential impact if *P*. *argentipes* are subjected to suboptimal levels of pyrethroid exposure, or additional resistance mechanisms evolve. The assays to detect *kdr* frequency changes provide a sensitive, high-throughput monitoring tool to detecting spatial and temporal variation in resistance in *P*. *argentipes*.

## Author summary

Visceral leishmaniasis is a fatal disease transmitted solely by the sandfly *Phlebotomus argentipes* in India. For decades, indoor residual spraying targeting sandflies with DDT has been the main control tool in the region. Emergence of DDT resistance has compromised this strategy and pyrethroids are now being implemented as alternative. Here, we describe the first molecular markers in sandflies for DDT and pyrethroid resistance. Two knockdown resistance (*kdr*) mutations in the *para* voltage-gated sodium channel target site gene are found at high frequency in natural populations in Bihar, the Indian focus of VL. The *kdr* variants strongly predict DDT resistance in *P*. *argentipes* and survival to lower-level pyrethroid exposure. Careful monitoring of resistance, aided by these markers, and of spraying efficacy is required to monitor changes and forewarn of emergent higher-level pyrethroid resistance in the region.

## Introduction

Visceral leishmaniasis (VL) causes over 20,000 deaths annually [1]. In the Indian subcontinent VL, also known as Kala-azar, is caused by the obligate intracellular protozoan *Leishmania donovani* that exhibits a continuous cycle comprising of humans, as the only known vertebrate host in the region, and females of the sandfly *Phlebotomus argentipes* [2]. Currently VL control and elimination strategies involve two main activities: rapid VL detection and treatment for humans and vector control of *P*. *argentipes* by indoor residual spraying (IRS) of neurotoxic insecticide [3]. In India, biannual DDT-based IRS has been a common sand fly control strategy in north-eastern regions where VL is most prevalent [4]. However, this control strategy has become compromised by the emergence of DDT resistance in *P*. *argentipes*; and quality of spraying is also a concern [4]. Surveillance studies using WHO assays have identified widespread DDT resistance in *P*. *argentipes* (mortality rates < 90% with DDT 4%), including in populations from regions with active VL transmission, notably in the state of Bihar [4–7]. Bihar is the main epicentre of VL transmission with 50% of the Indian subcontinent burden [8]. Visceral leishmaniasis has a higher incidence in densely-populated rural areas, particularly along the northern margins of the Ganges River, such as in Vaishali and neighbouring districts [9–12]. The elimination campaign has concentrated IRS in regions with higher VL incidence, spraying two times per year in houses and animal shelters. In 2015, prompted in part by DDT resistance in Vaishali and other northern districts such as Muzaffarpur and Samastipur [6,13], a switch to the pyrethroid alpha-cypermethrin was made for IRS. This scenario of VL incidence and DDT resistance contrasts with that found in Patna district along the southern margin of the Ganges River. Patna exhibits a lower VL incidence, particularly in urban areas of the state capital city [10], with a mosaic of DDT susceptible and resistant *P*. *argentipes* populations across villages, and IRS with DDT remains in use for control [13]. Currently there is a lack of knowledge of mechanisms underlying DDT resistance in *P*. *argentipes*, and phlebotomines generally, and how these might impact other insecticides, especially pyrethroids.

The major mechanisms of DDT resistance in insects are knockdown resistance mutations (*kdr*) within the *para* voltage-gated sodium channel gene in nerve cells (*Vgsc*) [14,15] and increased metabolism by glutathione S-transferases (GSTs) or, less commonly, cytochrome P450s [16,17]. Both DDT and pyrethroids target the insect VGSC protein. DDT increases sensitivity for depolarization of the channel, while pyrethroids only inhibit inactivation/deactivation processes of the channel; nevertheless each leads to stabilization of the open state and repetitive nerve firing, paralysis and ultimately death [18,19]. Multiple mutations in the *Vgsc* gene are linked to DDT and pyrethroid resistance in insects, particularly at codon 1014 (using *Musca domestica* codon numbering) [15]. Wild-type *Vgsc-*1014 is normally a leucine residue, and is located in the middle of hydrophobic segment S6 of domain II (IIS6) outside of the predicted binding domain [20]. Apparently contrary to this prediction, studies in cockroaches have indicated that *kdr* mutations in IIS6 may reduce binding affinity to insecticides [21]. Resistance might also arise via alternative mechanisms, such as changes in channel conformation and kinetics, indirectly affecting affinity between insecticides and their binding pockets [20,22]. Expression of insect *para*-VGSC in *Xenopus* oocytes has confirmed reduced sensitivity of the VGSC to insecticides for three *kdr-*1014 mutations (L1014F; L1014H and L1014S) though with substitution-specific impacts on different insecticides [23]. L1014F is the most common *kdr* mutation in insects, with L1014H also occurring across insect orders, whereas L1014S has only been found in mosquitoes to date [15], as have two other uncommon substitutions (L1014C [24] and L1014W [25]).

In the present study, a region of *Vgsc* in *P*. *argentipes* was sequenced and high-throughput SNP assays designed in order to: i) identify *kdr* mutations; ii) determine any association with resistance/tolerance to DDT and pyrethroids; and iii) assess their frequencies in natural populations of Bihar contrasting in VL incidence. We report the first resistance markers in a phlebotomine sandfly; with significant predictive utility as diagnostics to support sustainable insecticide use against *P*. *argentipes*.

## Methods

### Samples and insecticide bioassays

Weekly indoor resting collections of *P*. *argentipes* were carried out in central districts of Bihar state (India) between May and October 2015. Manual aspirators and torches were used to collect sand flies from inside human dwellings and cattle sheds during the day. Live sand flies were transported to the Rajendra Memorial Research Institute (RMRI) insectary, where blood fed and gravid *P*. *argentipes* females were placed into individual pots and maintained (at 28 ± 2°C; 70 ± 4% RH) until oviposition. Eggs were reared until the adult stage to obtain F1 adult samples. Further field collections were carried out using CDC light traps during the night in villages of two districts, Patna and Vaishali, between September and November 2015 (Table A in S1 Text). Sand flies were identified using morphological keys [26] and *P*. *argentipes* specimens stored at room temperature over silica gel. Householders provided informed consent for the collections, permission for which was granted by the ethical review boards of LSTM (protocol references 15.023, 15.036) and RMRI (reference 13/IEC/2015).

Bioassays followed the World Health Organization tube assay procedures [27] using papers impregnated with 4% DDT, 0.05% deltamethrin and 0.05% alpha-cypermethrin. Approximately 25 one day-old sugar-fed adult female *P*. *argentipes* were used in all bioassays. All sand flies were fed by cotton wool pad soaked with 10% sucrose. DDT and alpha-cypermethrin bioassays used the F1 progeny of wild caught females; bioassays with deltamethrin were carried out with adult females from a colony maintained at RMRI without previous insecticide exposure, though with occasional supplements from wild populations. No accepted standard exposure time exists for phlebotomines, but the mosquito standard of 60 min has previously proved appropriate for DDT [5] and was used for these bioassays. For the two pyrethroids, a 60 min exposure produced near ubiquitous mortality, therefore in order to produce some survivors for association-testing of mutations, pyrethroid exposure times were reduced to nominal times of 30 min for alpha-cypermethrin and 20 min for deltamethrin. Following exposure, sand flies were transferred to a holding tube and supplied with a cotton wool pad soaked with 10% sucrose. Mortality was recorded 24 hours after the bioassay. Each live female was classed as resistant to DDT or tolerant to pyrethroids (‘tolerant’ terminology is used to indicate survival, but to a shorter than standard exposure), or susceptible if dead after the recovery period. All sand flies were stored individually at room temperature over silica gel.

### Identification and genotyping of mutations in the voltage-gated sodium channel

Sequence information of the voltage-gated sodium channel gene (*Vgsc*) was obtained from genomes of two phlebotomine species, *Lutzomyia longipalpis* (VectorBase: *Lutzomyia longipalpis* Jacobina, LlonJ1.2) and *Phlebotomus papatasi* (VectorBase: *P*. *papatasi* Israel, PpapI1.2). The *Vgsc* sequences from these two species were used to design the conserved primers Vssc8F (5’–AATGTGGGATTGCATGCTGG–3’) and Vssc1bR (5’–CGTATCATTGTCTGCAGTTGGT–3’), which amplify a genomic DNA fragment from VGSC domain II, segment 6. This fragment included codon 1014 and other codons associated with insecticide resistance in *Aedes aegypti* (codons 1011 and 1016) and in Lepidoptera and Blattodea (codon 1020).

DNA extraction from individual *P*. *argentipes* was performed using the ChargeSwitch Forensic DNA Purification Kit (Thermo Fisher Scientific, MA, USA). Each amplification was performed separately in a 50 μl PCR reaction that contained 10X DreamTaq Green reaction buffer (Thermo Fisher Scientific), 2 mM MgCl_2_, 0.20 mM of each dNTP, 0.20 μM of each primer and 1U of DreamTaq DNA polymerase (Thermo Fisher Scientific). Thermocycling conditions included an initial denaturation step of 5 min at 95°C followed by 30 cycles each of 96°C for 30 s, 56°C for 30 s, and 72°C for 30 s, and a final extension step of 72°C for 5 min. The PCR products were purified with the QIAquick PCR Purification kit (Qiagen) and sequenced (in forward and reverse directions) using the same primers. Sequences were aligned using Codon Code Aligner version 4.2.7 (CodonCode Corporation, MA, USA).

From the sequence data, two TaqMan SNP genotyping assays (Thermo Fisher Scientific) were designed to identify four alleles detected at codon 1014 (see Results). The novel primer and probe sequences are provided in Table B in S1 Text. TaqMan reactions were performed in 10 μl volumes containing 1X SensiMix (Bioline, UK), 800 nM each primer, and 200 nM each probe on an Mx3005P qPCR thermal cycler (Agilent Technologies, CA, USA) with initial denaturation of 10 min at 95°C followed by 40 cycles of 15 s at 92°C and 1 min at 60°C.

### Data analysis

Abbott’s formula [28] was used to correct mortality rates of susceptibility bioassays with DDT, deltamethrin and alpha-cypermethrin. Chi-square tests or Fisher exact tests (where expected frequencies were low) were used to assess differences between resistant/tolerant and susceptible sand flies in allele and genotype frequencies at *Vgsc* codon 1014, with effect sizes measured using odds ratios. The proportion of each genotype group surviving was determined by dividing the number of live sand flies by the total number of sand flies per genotype. Lower and upper 95% confidence interval limits with correction for continuity were calculated for the survival proportions [29]. Multiple post-hoc pairwise comparisons used the Marascuilo and McSweeney method [30] (http://www.statstodo.com/MultiProp_Pgm.php) to identify significant differences between survival proportions of genotype groups. Sensitivity (ability of the test correctly identify resistant/tolerant individuals) and specificity (ability of the test correctly identify susceptible individuals) were calculated to verify the robustness of *Vgsc*-1014 assays to infer DDT resistance or pyrethroid tolerance in *P*. *argentipes*. Moreover, positive and negative predictive values for resistance/tolerance (probability that resistance/tolerance are presented for each *kdr* genotype group). Whenever multiple testing was performed, the nominal significance level of *α* = 0.05 was corrected by the sequential Bonferroni procedure [31].

### Accession numbers

Sequences were submitted to GenBank (Accession Numbers: KY114615-19).

## Results

### Detection and resistance association of *Vgsc* mutations

**Fig 1:**
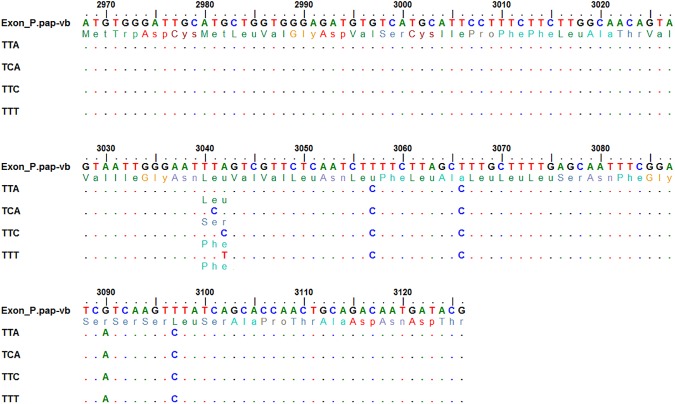
Sequence alignment of the *Vgsc* in *P*. *argentipes* with the sequence of *P*. *papatasi*. Nucleotide numbering is based on the house fly *para* sodium channel (Genbank accession number: X96668).

Bioassay mortalities were 43% with DDT (60 min exposure), 84% with alpha-cypermethrin (30 min exposure), and 56% with deltamethrin (20 min exposure) (Table C in S1 Text). A domain IIS6 fragment of the *Vgsc* gene was sequenced in a subsample of 48 *P*. *argentipes* (24 DDT resistant and 24 DDT susceptible). The wild-type leucine (L) codon (TTA) and three knockdown resistant polymorphisms were detected at codon 1014, comprising of a replacement of leucine with serine, L1014S (TCA) and two alleles (TTC and TTT) which each replace leucine with phenylalanine, L1014F (Fig 1 and Fig A in S2 Text; GenBank Accession Numbers: KY114615-19). Only wild-type sequences were identified at the other three codons within the fragment with previous association with insecticide resistance (i.e. 1011I/I, 1016V/V, and 1020F/F). No further non-synonymous mutations were detected within the IIS6 fragment sequenced. These sequences were used to design a pair of TaqMan SNP Genotyping Assays. The first assay differentiates two bases at the 2^nd^ nucleotide position of codon 1014 (TTA vs. TCA; Fig B in S2 Text), whereas the second assay differentiates the two alleles at the 3^rd^ nucleotide position (TTC vs. TTT; Fig C in S2 Text). Results of the assays are combined to define the 1014 genotype.

Allele frequencies at codon 1014 in females surviving or killed by each insecticide are shown in Table 1. For all insecticides there was no significant difference between survivorship for females possessing the two alternate phenylalanine alleles (TTC *vs*. TTT; Fisher exact test, minimum *P* = 0.13) and these were pooled for subsequent analyses. All comparisons between the *wt*-leucine allele and *kdr* alleles yielded significant differences associated with lower survival odds for the *wt*-leucine allele. Survival odds were significantly lower for 1014S than for 1014F for DDT, though not for either pyrethroid (Table 1).

**Table 1:** Allele frequencies at *Vgsc*-1014 and their relationship with insecticide susceptibility.

	*N*	Leu	Ser	Phe	Ser *vs*. Leu	Phe *vs*. Leu	Phe *vs*. Ser
		TTA	TCA	TTT/TTC			
DDT alive	166	12	93	61	OR = 9.35	OR = 26.32	OR = 2.79
		(7.3)	(56.0)	(36.7)			
DDT dead	166	82	68	16	*P =* 8 x 10^−13^	*P =* 2 x 10^−19^	*P =* 0.0013
		(49.4)	(41.0)	(9.6)			
delta alive	94	11	40	43	OR = 3.47	OR = 4.33	OR = 1.25
		(11.7)	(42.6)	(45.7)			
delta dead	124	42	44	38	*P =* 0.002	*P =* 0.0003	*P =* 0.54
		(33.9)	(35.5)	(30.6)			
alpha alive	46	3	34	9	OR = 23.81	OR = 35.71	OR = 1.47
		(6.5)	(73.9)	(19.5)			
alpha dead	274	176	83	15	*P =* 2 x 10^−12^	*P =* 2 x 10^−7^	*P =* 0.47
		(64.2)	(30.3)	(5.5)			

delta = deltamethrin; alpha = alpha-cypermethrin; *N*: number of alleles per group. Values in brackets are percentage relative frequencies within each group. OR = odds ratio for survival; *P*-values are from Fisher exact tests.

Based on a null hypothesis of recessivity for each *kdr* allele alone, but some degree of additivity when alternate *kdr* alleles are present as heterozygotes, the ten possible genotypes at codon 1014 were divided into four groups (Table 2). Low, and homogeneous survival among Leu homozygote and Leu/(Ser or Phe) heterozygote genotypes for DDT and alpha-cypermethrin exposure was concordant with the hypothesis of recessivity, although for deltamethrin Leu/Phe heterozygotes exhibited significantly lower mortality (Table 2). Genotype group frequencies differed strongly between surviving and dead sand flies for each insecticide (DDT: χ^2^_3_ = 75.2, *P* = 3 x 10^−16^; delta: χ^2^_3_ = 14.9, *P* = 0.002; alpha: χ^2^_3_ = 62.6, *P* = 2 x 10^−13^; Table 2), and the frequency of leucine-containing genotypes was much higher among dead sand flies (58%-87%) than in survivors (11%-23%). All *kdr*-only-genotype frequencies, whether homozygote or Ser/Phe heterozygotes, were higher in survivors than dead groups, supporting the hypothesis of additivity of alternate *kdr* alleles (Table 2).

**Table 2:** Genotype frequencies at *Vgsc*-1014 and their relationship with insecticide susceptibility.

		Leu/*				Ser/Ser	Ser/Phe	Phe/Phe
	*N*	Leu/Leu	Leu/Ser	Leu/Phe	total			
DDT alive	83	3	4	2	9	28	33	13
					(10.8)	(33.7)	(39.8)	(15.7)
DDT dead	83	20	32	10	62	15	6	0
					(74.7)	(18.1)	(7.2)	(0.0)
delta alive	47	0	0	11	11	11	18	7
					(23.4)	(23.4)	(38.3)	(14.9)
delta dead	62	6	12	18	36	8	16	2
					(58.1)	(12.9)	(25.8)	(3.2)
alpha alive	23	0	3	0	3	13	5	2
					(13.1)	(56.5)	(21.7)	(8.7)
alpha dead	137	57	51	11	119	15	2	1
					(86.9)	(10.9)	(1.5)	(0.7)

*N*: number of genotypes; Leu/*: number of genotypes that include at least one wild type leucine allele. Grey cells indicate significant variation in survival among Leu/* genotypes (*P* < 0.05 in Fisher exact test). Values in brackets are percentage relative frequencies.

Survivorship for each genotype group is summarised in Fig D in S2 Text and Fig E in S2 Text. Leucine genotypes exhibited consistently lower survival rates than *kdr-*only genotypes for DDT and alpha-cypermethrin (Table D in S1 Text, Fig D in S2 Text). For deltamethrin, significant variation in survival was only found between leucine genotypes and phenylalanine homozygotes (Table D in S1 Text), as a result of significantly higher survival rates of Leu/Phe genotypes than other leucine genotypes (Marascuilo post-hoc analysis, *P* = 0.0014; Fig D in S2 Text). When these Leu/Phe genotypes are removed from the leucine genotype group, a survival pattern more similar to that for DDT and alpha-cypermethrin is observed (Table D in S1 Text, Fig E in S2 Text).

**Fig 2:**
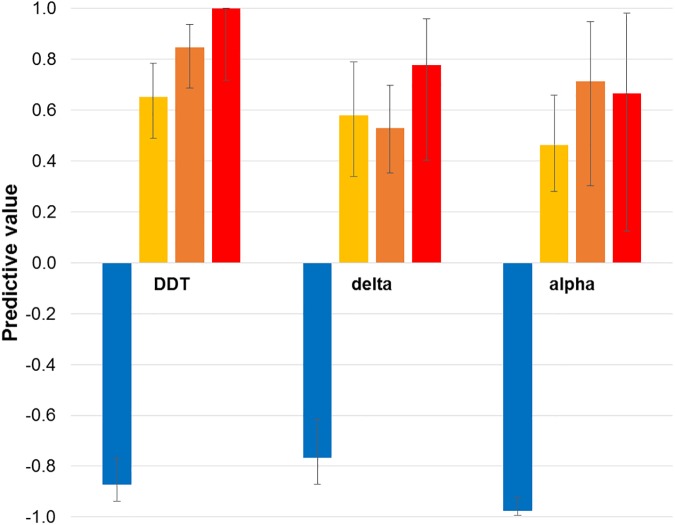
Positive and negative predictive values for DDT resistance and pyrethroid tolerance for each *Vgsc*-1014 genotype group. delta = deltamethrin; alpha = alpha-cypermethrin. Error bars are 95% confidence intervals.

The *Vgsc*-1014 assay gives generally (though not significantly) higher sensitivity (correct identification as resistant/tolerant) than specificity (correct identification as susceptible) (Table 3). Highest sensitivity is for identification of DDT resistance, and the most consistent values of sensitivity and specificity are for alpha-cypermethrin tolerance. Significantly lower specificity was evident for deltamethrin exposure (Table 3), with the assay suffering from grouping of leucine genotypes differing in resistance association (Table 2; Fig D in S2 Text, Fig E in S2 Text). Predictive values for individual genotype groups were typically high (Fig 2), especially for prediction of susceptibility (negative predictive values). Positive predictive values for DDT resistance exceeded 80% for both phenylalanine genotypes (Ser/Phe, Phe/Phe), while predictive values for pyrethroid tolerance ranged between 53% and 78% for these genotypes. Predictive values for resistance/tolerance are the lowest in serine homozygotes (47%–65%) for all insecticides (Fig 2).

**Table 3:** Sensitivity and specificity of the *Vgsc*-1014 assay for DDT resistance and pyrethroid tolerance in *P*. *argentipes*.

	TP	FP	TN	FN	sensitivity (%)	CI (%)	specificity (%)	CI (%)
DDT	74	21	62	9	89.2	80–95	74.7	64–84
delta	36	26	36	11	76.6	62–88	58.1	45–70
alpha	20	18	119	3	87.0	66–97	86.9	80–92

delta = deltamethrin; alpha = alpha-cypermethrin. TP = true positive (alive and *kdr*-only genotype); FP = false positive (dead and *kdr*-only genotype); TN = true negative (dead and *wt*-leucine genotypes); FN = false negative (alive and *wt*-leucine genotypes); sensibility = TP/(TP+FN); specificity = TN/(TN+FP); CI = 95% confidence interval limits.

### Inter-district variation in *kdr* mutation frequencies

**Fig 3:**
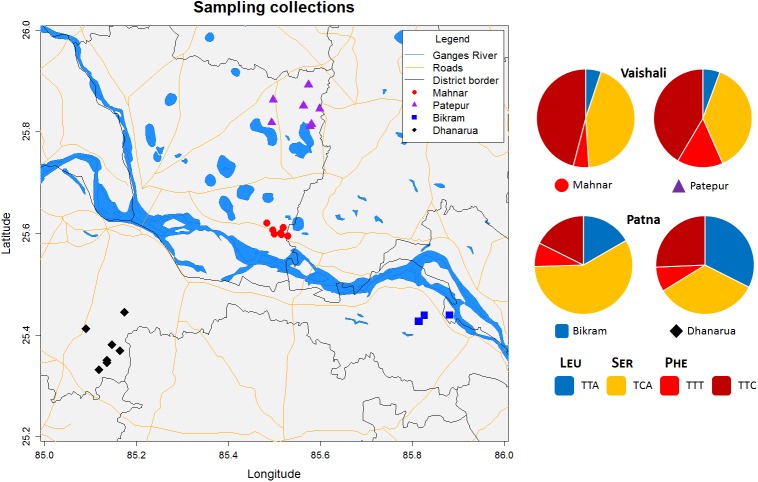
Primary health centre (PHC) collection locations and *Vgsc*-1014 allele frequencies within Vaishali and Patna districts (north and south of River Ganges, respectively). Map displayed by the R-package “maps” with shapefiles stored in DIVA GIS website (http://www.diva-gis.org/gdata).

In order to determine whether allele frequencies at codon 1014 varied on scales relevant to local control we genotyped samples from across Patna and Vaishali districts (Fig 3). Variation within districts was relatively limited, with no differences between the two PHCs in Vaishali (χ^2^_2_ = 0.80, *P* = 0.67); though a significant difference was present within Patna (χ^2^_2_ = 10.8, *P* = 0.005) primarily attributable to heterogeneity in serine allele frequency (Fig 3 and Table E in S1 Text). Intra-district comparisons of genotypic frequencies did not show any significant differences (Patna: χ^2^_3_ = 5.77, *P* = 0.12; Vaishali: χ^2^_3_ = 0.53, *P* = 0.91; Table F in S1 Text).

Both allelic and genotypic comparisons between Patna and Vaishali districts were highly significant (allelic χ^2^_2_ = 37.1, *P* = 9 x 10^−9^; genotypic: χ^2^_3_ = 21.4, *P* = 9 x 10^−5^). Vaishali exhibited approximately 10% wild type leucine genotypes, with seven of the 14 villages sampled entirely lacking the susceptible allele, and approximately 70% of all samples comprised of phenylalanine genotypes (Table A in S1 Text, Table E in S1 Text, and Table F in S1 Text). In contrast, Patna exhibited a threefold higher frequency of leucine genotypes and a lower proportion of phenylalanine genotypes (44%; Table A in S1 Text and Table F in S1 Text). These frequency data suggest a higher resistance to DDT and higher tolerance to pyrethroids in the more VL-affected Vaishali district than in Patna.

## Discussion

To our knowledge, this is the first detection of resistance mutations in phlebotomine sandflies and adds significantly to the very limited body of knowledge on insecticide resistance mechanisms. Previous work has detected elevated esterase and acetylcholinesterase activity in Sri Lankan *P*. *argentipes* [32] and the latter in *P*. *papatasi* from Sudan [33], but without clear links to insecticide resistance. Sequencing of a fragment of the *para* gene in *Lutzomyia longipalpis* from Brazil detected non-synonymous variants, though the amino acid polymorphisms have not be linked with resistance in any previous studies [34,35]. In contrast, the three polymorphisms we identified in *P*. *argentipes* code for two of the most frequently detected *kdr* mutations, L1014F and L1014S. Whilst L1014F is found across several insect orders, L1014S has only been found in the culicidae (*i*.*e*. *Anopheles* and *Culex*), in which it is very common in mosquitoes with a TTA leucine codon as the wild-type allele [14,15]. L1014F is linked with lower voltage sensitivity, which requires a stronger depolarization to open the channel, whereas serine 1014 promotes a faster inactivation of the channel without changing the activation sensitivity [23]. Significant associations were found between sandfly survival following exposure to DDT and the two class II pyrethroids tested and both *kdr-*1014 variants. Moreover, the effect of L1014F on DDT survival was significantly greater than L1014S. For both DDT and alpha-cypermethrin, the effect sizes (measured by odds ratios) were large and at the higher end of the spectrum of estimates from *Anopheles* [36]. For deltamethrin the quantitative estimates of effect size are somewhat lower but should be interpreted more cautiously owing to the use of laboratory colony sand flies, rather than the F1 near-wild females used for the other insecticide bioassays.

The effects of each mutation seem to be partially or fully recessive. Heterozygotes with a *wt*-leucine exhibited similar tolerance to insecticide as *wt*-leucine homozygotes (survival rates << 0.4) with the exception of Leu/Phe heterozygotes for deltamethrin (survival rate ≈ 0.4). This largely recessive nature of the *kdr* mutations in *P*. *argentipes* is consistent with previous knowledge in mosquitoes and other dipterans [37–39]. For DDT we found significant differences between the moderate resistance of serine homozygotes (survival rate ≈ 0.65) and the strong resistance of phenylalanine homozygotes (100% survival rate). Heterozygotes possessing both *kdr* variants showed an intermediate survival rate suggesting some level of additivity of the alternate *kdr* alleles and a mechanism to overcome recessivity of the individual mutations. Such a stronger resistant phenotype in 1014S when paired with 1014F has also been reported (for the same two 1014 mutations) in African *Anopheles* mosquitoes [40]. Differences between the associations of the two *kdr* mutations were less clear, and not significant, for the two pyrethroids. For alpha-cypermethrin, *kdr-*only genotypes (excluding phenylalanine homozygotes due to low statistical power) were consistently different from the susceptible *wt*-leucine genotypes, but serine homozygotes exhibited a survival rate below 50% suggesting a weaker tolerance phenotype than for phenylalanine. For deltamethrin, only phenylalanine homozygotes were significantly different from *wt*-leucine genotypes, but heterogeneity among *wt*-leucine genotypes reduced power. Overall, results suggest that phenylalanine is likely to yield a stronger tolerance to class II pyrethroids than serine, which is consistent with expression assays of *Drosophila* VGSC in *Xenopus* oocytes [23] and data from *Anopheles* field populations [40]. Further studies will be necessary to confirm this hypothesis in *P*. *argentipes* under test conditions using established diagnostic doses.

Though L1014F seems to confer a stronger resistant/tolerant phenotype, L1014S may offer other advantages in the field by reducing potential fitness costs that are normally associated with L1014F “less excitable channels” [23,41]. Moreover, indirect costs may also emerge as a by-product of strong positive selection from insecticide resistance by reducing linked genetic diversity in neighbouring genomic regions of the *para* gene, which may interfere with other selection processes at nearby beneficial mutations [42]; for example in *L*. *longipalpis*, variation in the *para* gene is also associated with courtship song variation [34]. Fitness costs could reduce the viability of L1014F homozygotes and potentially increase the benefit of heterozygosity in natural populations, particularly in the absence of insecticide pressure. Nevertheless, our study regions have active control programs and significant deviations from Hardy-Weinberg equilibrium were absent from *Vgsc-*1014 (Bikram, Patna district; Vaishali district) or where detected, associated with a low proportion of leucine, rather than an excess of 1014F/S heterozygotes (Dhanarua, Patna district; Table G in S1 Text). These results suggest that any potential fitness costs associated with L1014F are currently being overcome by strong selective pressures, a hypothesis consistent with observation of significantly higher 1014F frequencies in Vaishali, which is subjected to greater IRS. Further studies are required to determine whether a reduction in DDT selection pressure may lead to an alteration in the balance of 1014F/S/L in *P*. *argentipes* and how *kdr* mutations in other regions of the sodium channel (e.g. IIS5, IIIS6) may be implicated in DDT/pyrethroid resistance of *P*. *argentipes*.

DDT resistance is now well described and established in *P*. *argentipes* from north-eastern India. This contrasts with an apparent lack of pyrethroid resistance in the region [4–7,13], though with the important caveat that accepted WHO diagnostic doses for definition of resistance have yet to be ascertained for *Phlebotomus* spp. Until very recently, DDT has been the main choice of Indian control programs since the 1950s, while a slightly earlier switch to pyrethroids was made by the VL control programs in Bangladesh and Nepal [43,44]. Long-term use of DDT presents a strong selective pressure for resistance and temporal analysis suggests an increase over time [4]. Capacity for populations to evolve resistance is also likely to be enhanced if sub-lethal concentrations are sprayed or maintained in the environment after spraying [4]. Given this long-term use of DDT it is perhaps surprising that *kdr* mutations provide such a strong prediction of resistance phenotype, because over time additional resistance mechanisms would be expected to develop [45]. Nevertheless, it is plausible that other forms of resistance mechanism may contribute to higher level resistance, a hypothesis of high importance for control that is currently under investigation.

## Conclusion

Natural populations of *P*. *argentipes* in central Bihar have high frequencies of *kdr-*1014 mutations, particularly in Vaishali where in several villages *wt-*leucine alleles were absent from our samples suggesting possible fixation of *kdr* mutations; now, or in the near future. The high proportion of L1014F is consistent with insecticide bioassays that show high DDT resistance in most villages in Vaishali [13]. Bioassays for *P*. *argentipes* are very challenging to execute on a large scale because larvae are extremely difficult to collect and rearing of F1s is time-consuming and technically difficult. Monitoring of *kdr*-1014 mutations by molecular assays may thus provide a rapid, high throughput tool that does not require live sand flies to infer DDT resistance across *P*. *argentipes* populations when compared with bioassays (e.g. WHO cone and tube tests). DNA diagnostics provide greater flexibility in study methodology, allowing screening of stored samples (e.g. permitting a temporal gap between collection and analysis) and simplifying sampling for control programs that may potentiate wider geographic collections. The high proportion of L1014F in Vaishali could also bring challenges, linked to the higher tolerance to pyrethroids associated with *kdr-*1014 mutations. This may potentiate the emergence of problematic pyrethroid resistance by providing a foundation for resistance upon which other mechanisms can develop [36]. For this reason, it is important to evaluate insecticides with alternative target sites, such as acetylcholinesterase inhibitors (organophosphates and carbamates) that may be integrated into an IRS rotation system.

## Supporting information

S1 Text Supplemental tables. (DOCX)

S2 Text Supplemental figures. (DOCX)
