# Supplementary material for: Knockdown resistance mutations predict DDT resistance and pyrethroid tolerance in the visceral leishmaniasis vector Phlebotomus argentipes
Source: PLoS Negl Trop Dis. 2017 Apr 17;11(4):e0005504. doi: 10.1371/journal.pntd.0005504 (PMC5407848; doi:10.1371/journal.pntd.0005504)
Supplement: S1 Text — (DOCX) [file pntd.0005504.s001.docx]

**Table A** – Geographical information for sampling sites and frequencies of *Vgsc-*1014 alleles in each village.

| **District** | **PHC** | **Village** | **latitude** | **longitude** | ***N*** | **Leu** | **Ser** | **Phe** |
| --- | --- | --- | --- | --- | --- | --- | --- | --- |
| Vaishali | Mahnar | Hasanpur North | 25.594133 | 85.529850 | 18 | 0.111 | 0.444 | 0.444 |
|  |  | Alipur Hatta | 25.597633 | 85.514733 | 20 | - | 0.350 | 0.650 |
|  |  | W.No. 01 to 11 | 25.619700 | 85.483733 | 18 | - | 0.667 | 0.333 |
|  |  | Lawapur Narayan | 25.600650 | 85.514550 | 14 | 0.071 | 0.357 | 0.571 |
|  |  | W.No. 18 to 23 | 25.606733 | 85.496967 | 8 | - | 0.500 | 0.500 |
|  |  | Chakkeso | 25.598133 | 85.499950 | 2 | - | 0.500 | 0.500 |
|  |  | Musapur | 25.611483 | 85.519050 | 18 | 0.111 | 0.333 | 0.556 |
|  | Patepur | Asama | 25.818000 | 85.493749 | 10 | 0.200 | 0.200 | 0.600 |
|  |  | Sakrauli | 25.810717 | 85.577150 | 8 | - | 0.500 | 0.500 |
|  |  | Nirpur Bagh | 25.814400 | 85.581000 | 10 | - | 0.100 | 0.900 |
|  |  | Bahuara | 25.845233 | 85.598267 | 8 | 0.125 | 0.375 | 0.500 |
|  |  | Baligaon | 25.891800 | 85.573833 | 10 | - | 0.400 | 0.600 |
|  |  | Kusahi Nirpur | 25.850683 | 85.562900 | 18 | 0.056 | 0.389 | 0.556 |
|  |  | Ramauli | 25.862717 | 85.497233 | 42 | 0.048 | 0.452 | 0.500 |
| Patna | Bikram | Azad Nagar | 25.438867 | 85.826633 | 26 | 0.038 | 0.615 | 0.346 |
|  |  | Parriyanwa | 25.438300 | 85.826567 | 24 | - | 0.625 | 0.375 |
|  |  | Raghunathpur | 25.426867 | 85.813367 | 22 | 0.409 | 0.545 | 0.045 |
|  |  | Kanpa | 25.426700 | 85.814900 | 20 | 0.250 | 0.500 | 0.250 |
|  |  | Masudha Telpa | 25.439150 | 85.881350 | 10 | 0.200 | 0.600 | 0.200 |
|  | Dhanarua | Kaili | 25.345467 | 85.135267 | 12 | - | 0.250 | 0.750 |
|  |  | Morriyawan | 25.350500 | 85.135267 | 10 | - | 0.300 | 0.700 |
|  |  | Harla | 25.332303 | 85.118000 | 10 | 0.200 | 0.500 | 0.300 |
|  |  | Beldari chak | 25.444783 | 85.174217 | 8 | 0.125 | 0.625 | 0.250 |
|  |  | Tetri | 25.412517 | 85.090233 | 4 | - | 0.500 | 0.500 |
|  |  | Jahanpur | 25.369950 | 85.163417 | 26 | 0.654 | 0.269 | 0.077 |
|  |  | Bakhari | 25.381300 | 85.146417 | 4 | 1.000 | - | - |

PHC: primary health centre; a smaller geographic division within districts. *N*: total number of alleles in each village; Leucine: wild type allele.

**Table B** – Primers and probes for TaqMan® SNP Genotyping Assays for *Vgsc-*1014 alleles.

| Mutation | Primer name | Sequence | Primer name | Sequence | Codon | Probe name | Dye | Sequence |
| --- | --- | --- | --- | --- | --- | --- | --- | --- |
| Pa_1_C_T  (2^nd^ position) | Pa_1_C_T_F | CATTCCTTTCTTCTTGGCAACAGT | Pa_1_C_T_R | TCCATAGTCACTGTGTGTTGAAAATCA | TTA (L) | Pa_1_C_T_V | VIC | TCACGACTAAATTCC |
|  |  |  |  |  | TCA (S) | Pa_1_C_T_M | FAM | ACGACTGAATTCC |
| Pa_2_T_C  (3^rd^ position) | Pa_2_T_C_F | CATTCCTTTCTTCTTGGCAACAGT | Pa_2_T_C_R | CGTCCATAGTCACTGTATGTTGAAAATCA | TTT (P) | Pa_2_T_C_V | VIC | TCACGACAAAATTC |
|  |  |  |  |  | TTC (P) | Pa_2_T_C_M | FAM | ACGACGAAATTC |

**Table C** – Susceptibility bioassay results for three insecticides in *P. argentipes*.

|  | Total | dead | alive | Total control | alive control | % corrected mortality^a^ |
| --- | --- | --- | --- | --- | --- | --- |
| DDT 4% (60’) | 187 | 98 | 89 | 85 | 71 | 43.0 |
| Deltamethrin 0.05% (20’) | 109 | 62 | 47 | 65 | 64 | 56.2 |
| Alpha-cypermethin 0.05% (30’) | 167 | 140 | 27 | 66 | 65 | 83.6 |

^a^mortality corrected using Abbott’s method.

**Table D –** Results from Marascuilo's pairwise *post hoc* analysis comparing four groups.

|  | Leu/* *vs.* Ser/Ser | Leu/* *vs.* Ser/Phe | Leu/* *vs.* Phe/Phe | Ser/Ser *vs.* Ser/Phe | Ser/Ser *vs.* Phe/Phe | Ser/Phe *vs.* Phe/Phe |
| --- | --- | --- | --- | --- | --- | --- |
| DDT | ***P* <0.0001** | ***P* <0.0001** | ***P* <0.0001** | *P =* 0.220 | ***P* <0.0001** | *P =* 0.069 |
| Delta | *P =* 0.067 | *P =* 0.049 | ***P =* 0.005** | *P =* 0.989 | *P =* 0.745 | *P =* 0.508 |
| Delta (excluding Leu/Phe group) | ***P* <0.0001** | ***P* <0.0001** | ***P* <0.0001** | *P =* 0.998 | *P =* 0.873 | *P =* 0.676 |
| Alpha | ***P* <0.0001** | ***P =* 0.001** | *P =* 0.136 | *P =* 0.650 | *P =* 0.920 | *P* = 0.999 |

Delta: Deltamethrin; Alpha: Alpha-cypermethrin; Leu/*: number of genotypes that include at list one wild type leucine allele. Significant values are shown in bold.

**Table E** – Frequency of *Vgsc*-1014 alleles by PHC/District.

| PHC/District | *N* | Leucine | L1014S |  | L1014F |  |
| --- | --- | --- | --- | --- | --- | --- |
|  |  | TTA | TCA | TTT/TTC | TTT | TTC |
| Bikram | 102 | 17  (16.7) | 59  (57.8) | 26  (25.5) | 8  (7.8) | 18  (17.6) |
| Dhanarua | 74 | 24  (32.4) | 25  (33.8) | 25  (33.8) | 6  (8.1) | 19  (25.7) |
| Patna District | 176 | 41  (23.3) | 84  (47.7) | 51  (29.0) | 14  (8.0) | 37  (21.0) |
| Mahnar | 98 | 5  (5.1) | 43  (43.9) | 50  (51.0) | 5  (5.1) | 45  (45.9) |
| Patepur | 106 | 6  (5.7) | 40  (37.7) | 60  (56.6) | 16  (15.1) | 44  (41.5) |
| Vaishali District | 204 | 11  (5.4) | 83  (40.7) | 110  (53.9) | 21  (10.3) | 89  (43.6) |

*N:* number of alleles per PHC/District. Values in brackets represent relative frequencies (in percentage). Leucine is *Vgsc* wild type; L1014S and L1014F are amino acid polymorphisms. TTT/TTC are separate nucleotide variants which both lead to the phenylalanine mutant, therefore the TTT/TTC column is the sum of the individual TTT and TTC columns.

**Table F** – Genotype frequencies of *Vgsc*-1014 by PHC/District.

| PHC/District | *N* | Leu/* | | | | Ser/Ser | Ser/Phe | Phe/Phe |
| --- | --- | --- | --- | --- | --- | --- | --- | --- |
|  |  | Leu/  Leu | Leu/  Ser | Leu/  Phe | total |  |  |  |
| Bikram | 51 | 3 | 8 | 3 | 14  (27.5) | 19  (37.3) | 13  (25.5) | 5  (9.8) |
| Dhanarua | 37 | 9 | 4 | 2 | 15  (40.5) | 6  (16.2) | 9  (24.3) | 7  (18.9) |
| Patna District | 88 | 12 | 12 | 5 | 29  (33.0) | 25  (28.4) | 22  (25.0) | 12  (13.6) |
| Mahnar | 49 | 0 | 2 | 3 | 5  (10.2) | 11  (22.4) | 19  (38.8) | 14  (28.6) |
| Patepur | 53 | 0 | 1 | 5 | 6  (11.3) | 9  (17.0) | 21  (39.6) | 17  (32.1) |
| Vaishali District | 102 | 0 | 3 | 8 | 11  (10.8) | 20  (19.6) | 40  (39.2) | 31  (30.4) |

Leu/*: number of genotypes that include at list one wild type leucine allele (i.e. are insecticide-susceptible); Ser/Ser: number of individuals homozygous for serine; Ser/Phe: number of heterozygotes with serine and phenylalanine; Phe/Phe: number of homozygotes for phenylalanine. Values in brackets are relative frequencies (percentage).

**Table G** – Tests for Hardy-Weinberg equilibrium for the *Vgsc* variants in each PHC.

| PHC | Allelic frequency | | | Leu/Ser | | Leu/Phe | | Ser/Phe | | HW |
| --- | --- | --- | --- | --- | --- | --- | --- | --- | --- | --- |
|  | Leu | Ser | Phe | H_E_ | H_O_ | H_E_ | H_O_ | H_E_ | H_O_ |  |
| Bikram | 0.167 | 0.578 | 0.255 | 0.193 | 0.157 | 0.085 | 0.059 | 0.295 | 0.255 | *P* = 0.228 |
| Dhanarua | 0.324 | 0.338 | 0.338 | 0.219 | 0.108 | 0.219 | 0.054 | 0.228 | 0.243 | ***P* = 0.0006** |
| Mahnar | 0.051 | 0.439 | 0.510 | 0.045 | 0.041 | 0.052 | 0.061 | 0.448 | 0.388 | *P* = 0.640 |
| Patepur | 0.057 | 0.377 | 0.566 | 0.043 | 0.019 | 0.065 | 0.094 | 0.427 | 0.396 | *P* = 0.532 |

H_E_: Expected Heterozygosity; H_O_: Observed Heterozygosity; HW: Tests for Hardy-Weinberg equilibrium. Significant values are shown in bold.
