# Supplementary material for: Knockdown resistance mutations predict DDT resistance and pyrethroid tolerance in the visceral leishmaniasis vector Phlebotomus argentipes
Source: PLoS Negl Trop Dis. 2017 Apr 17;11(4):e0005504. doi: 10.1371/journal.pntd.0005504 (PMC5407848; doi:10.1371/journal.pntd.0005504)
Supplement: S2 Text — (DOCX) [file pntd.0005504.s002.docx]

***
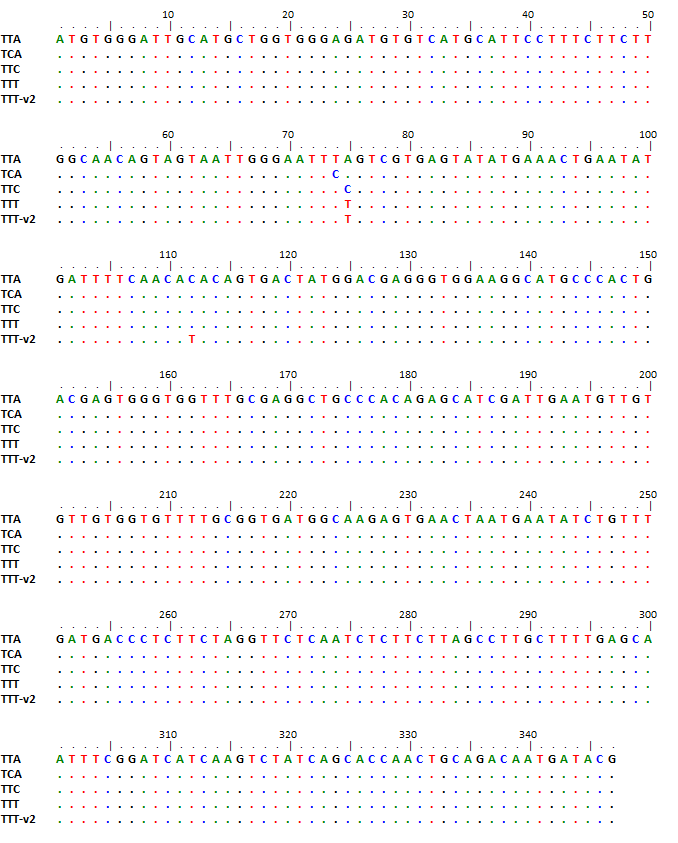
***

**Fig A** – Alignment of the sequences obtained from the domain IIS6 fragment of *Vgsc* in *P. argentipes.*

***
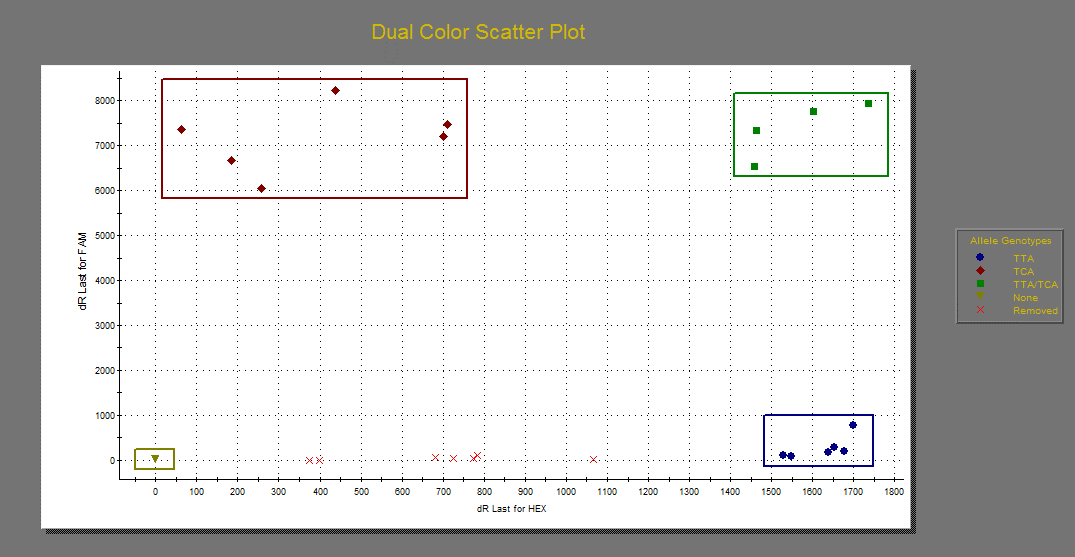
***

**Fig B** – Representative endpoint scatter plot for TaqMan SNP genotyping assay at 2^nd^ nucleotide position, TTA (VIC) *vs*. TCA (FAM).

Crosses show ‘removed’ points indicating non-target binding of the TTA (HEX; x -axis) probe to TTT and TTC alleles (where a null ‘None’ result should occur). Since, both assays are interpreted simultaneously, results are unambiguous (see Fig C).

**
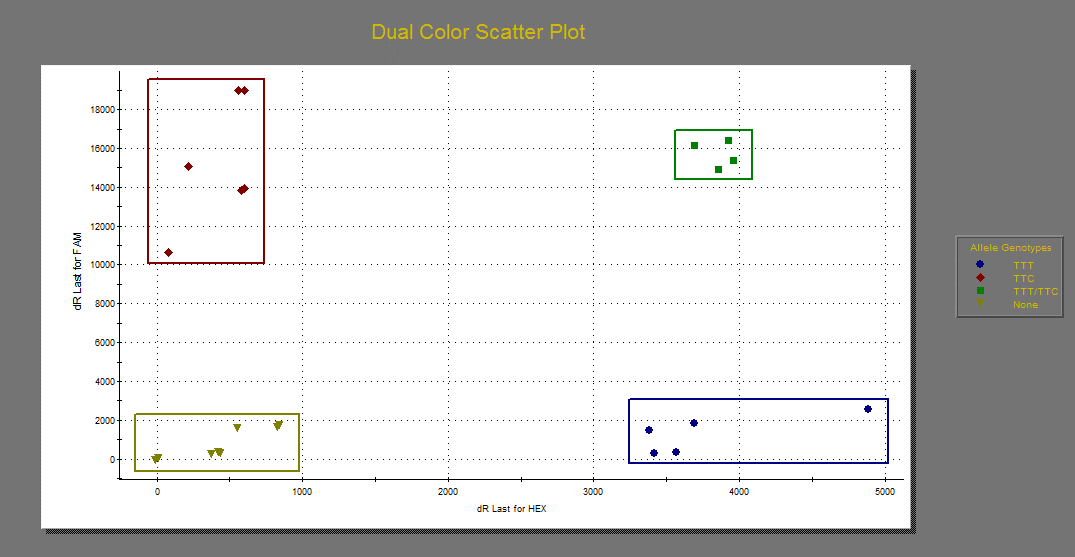
**

**Fig C** – Representative endpoint scatter plot for TaqMan SNP genotyping assay at 3^rd^ nucleotide position, TTT (VIC) *vs*. TTC (FAM).

**Fig D** – Survival proportion by insecticide for wild type genotypes (Leu*, which contain at least one leucine allele) and three *kdr/kdr* genotype groups.

Error bars are 95% confidence intervals. Pairwise *post hoc* test results are shown in Table D in S1 Text.

**Fig E** – Survival proportion by insecticide for five genotype groups with Leu/Phe shown separately (see Table 2).

Error bars are 95% confidence intervals. Pairwise *post hoc* test results are shown in Table D in S1 Text.
